# A Case for Systematic Quality Management in Mosquito Control Programmes in Europe

**DOI:** 10.3390/ijerph18073478

**Published:** 2021-03-27

**Authors:** Antonios Michaelakis, Fabrizio Balestrino, Norbert Becker, Romeo Bellini, Beniamino Caputo, Alessandra della Torre, Jordi Figuerola, Gregory L’Ambert, Dusan Petric, Vincent Robert, David Roiz, Anastasios Saratsis, Carla A. Sousa, William G. R. Wint, Nikos T. Papadopoulos

**Affiliations:** 1 Scientific Directorate of Entomology and Agricultural Zoology, Benaki Phytopathological Institute, 14561 Kifissia, Greece; a.michaelakis@bpi.gr; 2 Centro Agricoltura Ambiente “G. Nicoli”, 40014 Crevalcore, Italy; fbalestrino@caa.it (F.B.); rbellini@caa.it (R.B.); 3 German Mosquito Control Association (KABS), 67346 Speyer, Germany; norbertfbecker@web.de; 4 Dipartimento di Sanità Pubblica e Malattie Infettive, Università di Roma “Sapienza”, 00185 Rome, Italy; beniamino.caputo@uniroma1.it (B.C.); alessandra.dellatorre@uniroma1.it (A.d.T.); 5 Department of Wetland Ecology, Estación Biológica de Doñana, CSIC, Avenida Américo Vespucio 26, E-41092 Sevilla, Spain; jordi@ebd.csic.es; 6 CIBER Epidemiología y Salud Pública, 28029 Madrid, Spain; 7 EID Méditerranée, Division Research and Development, 34184 Montpellier, France; glambert@eid-med.org; 8 Department of Plant and Environment Protection, Faculty of Agriculture, University of Novi Sad, 21000 Novi Sad, Serbia; dusan.petric@polj.uns.ac.rs; 9 MIVEGEC, University Montpellier, IRD, CNRS, 34090 Montpellier, France; vincent.robert@ird.fr (V.R.); davidroiz@gmail.com (D.R.); 10 Laboratory of Parasitology, Veterinary Research Institute, Hellenic Agricultural Organisation Demeter, 57001 Thermi, Greece; saratsis@vri.gr; 11 Global Health and Tropical Medicine, Instituto de Higiene e Medicina Tropical, Universidade Nova de Lisboa, 1349-008 Lisboa, Portugal; CASousa@ihmt.unl.pt; 12 Environmental Research Group Oxford Ltd., c/o Department of Zoology, South Parks Road, Oxford OX1 3PS, UK; william.wint@zoo.ox.ac.uk; 13 Laboratory of Entomology and Agricultural Zoology, University of Thessaly, 38446 Volos, Greece

**Keywords:** Culicidae, pest management, invasion, arthropod vectors, vector borne diseases, nuisance, insecticides

## Abstract

The recent spread of invasive mosquito species, such as *Aedes albopictus* and the seasonal sporadic transmission of autochthonous cases of arboviral diseases (e.g., dengue, chikungunya, Zika) in temperate areas, such as Europe and North America, highlight the importance of effective mosquito-control interventions to reduce not only nuisance, but also major threats for public health. Local, regional, and even national mosquito control programs have been established in many countries and are executed on a seasonal basis by either public or private bodies. In order for these interventions to be worthwhile, funding authorities should ensure that mosquito control is (a) planned by competent scientific institutions addressing the local demands, (b) executed following the plan that is based on recommended and effective methods and strategies, (c) monitored regularly by checking the efficacy of the implemented actions, (d) evaluated against the set of targets, and (e) regularly improved according to the results of the monitoring. Adherence to these conditions can only be assured if a formal quality management system is adopted and enforced that ensures the transparency of effectiveness of the control operation. The current paper aims at defining the two components of this quality management system, quality assurance and quality control for mosquito control programs with special emphasis on Europe, but applicable over temperate areas.

## 1. Introduction

Anthropogenic changes at a local, regional and global scale, such as land use change, increasing trade, travel and urbanization, coupled with current and predicted climate changes, accelerate several processes that strongly enhance the incidence and spread of mosquito-borne diseases, not only in tropical but also in temperate areas [1,2]. In Europe, mosquito-borne diseases have recently re-emerged as a major public health threat, following two large outbreaks of chikungunya in Italy [3,4], additional sporadic cases of autochthonous dengue and chikungunya transmission by invasive *Aedes* species (i.e., *Aedes albopictus*) in Croatia, France, Italy and Spain [5,6,7], reoccurrence of *Plasmodium* parasites in Greece [8] and increasing transmission of endemic pathogens (i.e., West Nile virus) by native mosquito species (i.e., *Culex pipiens*) in several countries [9].

As a response to the public health threats posed by native and invasive mosquitoes and to the increasing nuisance due to aggressive day-biting *Aedes* invasive species, mosquito control programs are being increasingly implemented in Europe. They significantly vary with respect to the objective, target species, ecological situation, scale and available resources. They also exploit and integrate different approaches, from social engagement to reduce mosquito breeding sites, to use of larvicides (e.g., insect growth regulators (IGRs), or *Bacillus thuringiensis israelensis* (Bti)), to spraying of adulticide insecticides, to release of sterilized (SIT) or incompatible (IIT) males. Although a complete figure of the costs of public mosquito control in the European region is not available, it has been estimated that Greece invests approximately 7 million €/year, while Emilia-Romagna Region in Italy and the Upper Rhine Valley in Germany spend between 2–4 million €/year each [10,11,12,13]. It is thus safe to state that hundreds of millions of euros in taxes are devoted to mosquito control in EU every year.

In contrast to management of agricultural insect pests, whose benefit is measured in terms of increased yield, the benefit of protecting people from mosquito bites and increased wellbeing is challenging to quantify. In the majority of cases, private pest control operators (PCOs) selected through public tenders are responsible for the planning of the interventions and the selection of appropriate tools/approaches for an efficient implementation. Most PCOs are certified by the International Organization of Standardization (ISO), which defines only general standards to ensure the quality, safety, and efficiency of products, services, and systems, as well as their traceability. In some cases, public bodies may also undertake mosquito control operations. Whether these programs are cost-effective, efficient and appropriately implemented is challenging to determine. The European Centre of Disease Prevention and Control (ECDC) and the World Health Organization (WHO) stress the importance of careful monitoring of the quality of the mosquito control programs [14,15,16,17,18,19], which preferably should be implemented by an external body of experts to promote transparency. Nevertheless, such assessment is rarely carried out in Europe. This is confirmed by the results of a survey on current practices in mosquito surveillance and control carried out in the framework of the AIM-COST Action (see results of Question 16 in https://www.aedescost.eu/sites/default/files/2020-08/AIMcOSTQuestionnaire_Full_Report_postedV4.pdf (accessed on 25 March 2021).

This paper reflects the view of 15 experts (see authors list) on mosquito biology and control from seven EU countries—networking within the *Aedes* Invasive Mosquito (AIM) COST Action (CA17108; www.aedescost.eu; accessed on 25, March, 2021) framework. The above experts include academics, public officers, animal health officers, as well as representatives from public and private mosquito control companies. We argue that there is an urgency to advocate for external quality management (QM) in mosquito control programs in Europe. This paper summarizes procedures, methods and indicators for the implementation of the two QM components (i.e., quality assurance (QA) and quality control (QC)), highlights major challenges and constraints, and indicates who should take the responsibility for funding, planning and execution. The final aim is to induce stakeholders (e.g., academics, public and animal health officers, public and private mosquito control companies) to become involved in this crucial issue.

## 2. Quality Assurance and Quality Control in Mosquito Control Programs

**Figure 1:**
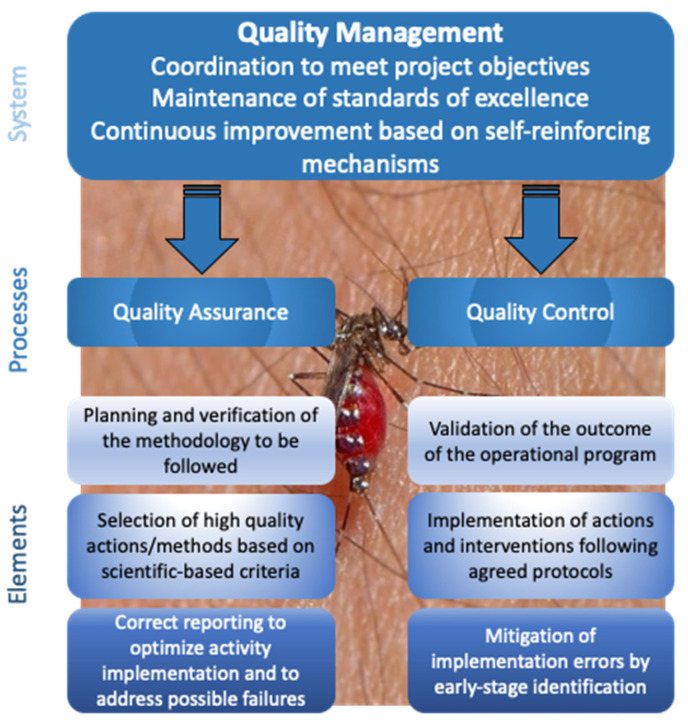
Schematic outline of quality management processes and elements (background: *Aedes albopictus* receiving blood meal from human arm, photo by Stihios Serafim).

Quality assurance (QA) and quality control (QC) processes are the two components of a QM system, which (a) coordinates and directs all activities, tasks and responsibilities needed to achieve the objectives of a mosquito control campaign; and (b) maintains standards of excellence and continuously improves its effectiveness and efficiency (Figure 1). Quality assurance refers to planning and verification of the methodology to be followed to meet the goals of the mosquito control program. The QA process ensures that all planned actions/methods have been selected based on scientific-based criteria and will be implemented correctly, in order to prevent possible failures, optimize execution of activities and assure accurate reporting. On the other hand, QC is a process that validates the outcome of the operational program, ensuring that the applied actions/methods are implemented appropriately, aiming to identify, reduce and even eliminate errors, validating the achievement of control objectives.

The QM system requires establishment of mutually agreed protocols between actors assigning and carrying out control programs. The objectives of mosquito control programs may vary according to the context and political will. For instance, eradication and/or containment might be the ultimate goal in the case of invasion events or recently established invasive mosquito species. On the other hand, routine (often calendar-based) interventions aim to reduce mosquito populations and hence decrease nuisance. In public health risk situations, interventions aim to prevent pathogen transmission, eradicate the disease or reduce the size of an epidemic. In any case, activities should be tailored to the size of the area to be treated, the type of land use and the target mosquito species.

Currently mosquito control is mainly based on integrated pest management, relying on the combined use of several mosquito control tools selected according to evidence provided by surveillance. The establishment of a consistent, reliable and sustainable long-term surveillance system is therefore a critical component to any successful mosquito control program in order to make informed decisions and respond appropriately to changing mosquito populations. Irrespective of the control methods planned, it is also essential to make sure that surveillance activities also conform to appropriate QM criteria. As with control, these will vary according to the surveillance methods used. The “assessment of the efficacy” must be considered as a deliverable of any mosquito control program and thus considered part of QC and under the umbrella of the QM process.

Table 1 lists major components of mosquito control programs that can be adopted in an integrated mosquito management strategy and their relative QA and QC best practices. This list includes either conventional strategies (i.e., adulticide and larvicide treatments, source reduction in public and private areas) and some other approaches that have been recently implemented or are believed to be most promising for *Ae. albopictus* control in European and other temperate regions.

## 3. Structure and Procedures of QA and QC Programs

The body responsible for QM should be independent (not related to the operating pest control actors). An independent evaluation within funding authorities is challenging and needs to be meticulously designed and operated. The external QM bodies could be appointed by the legal act of the country or chosen through the procurement procedure from the qualified public institutions (e.g., university, public health agency, research institute) or private companies qualified for QC and not engaged in mosquito control activities.

Considering the operational phase, actions and procedures, and dissemination level, the implementation of QA and QC in mosquito control programs involves four phases that are aligned with mosquito activities [20] (Table 2). These include (1) exploration, which determines the necessity to implement an organized mosquito control campaign; (2) planning, which encompasses all actions needed to get to the implementation phase; (3) selection of operating actors and processes with specific, successive implementation of interventions following the designed plan; (4) evaluation of the success of the operation and of the achievement of the expected goals. It is important to consider that these phases must be in accordance with the respective national regulatory and legislative frameworks, as set by their governance mechanisms.

The exploratory phase begins with receiving/filling a demand for a mosquito control program and the associated request for budget and resources. First the goal of the project, tightly connected to resources requested, should be defined. The need for control programs should be supported by epidemiological or surveillance data and risk or nuisance estimates. Public consultation as well as a scientific opinion are also necessary to make decisions on whether the project should be approved and supported or not. The decision-making process may often not be straightforward, involve several legal bodies and may take longer than expected. This has severe consequences for the whole program and may affect monitoring and efficacy assessment.

The planning phase might come within the respective municipality’s, region’s or both jurisdictions, depending on the country. It is the administrative authority’s task to engage scientific institution/experts in identifying the actions, objectives, desired outcomes and goals based on local needs as part of the mosquito control plan. This ideally should take into consideration information from local mosquito surveillance, environmental and public health data (in case of outbreaks) [17]. Due to the often-unprecedented nature of outbreaks, special care should be taken to include QM actions to be implemented in case of an epidemic. These plans should be reviewed, as part of a QA procedure, by an independent/external evaluator. The results of this evaluation, presented in the form of a report, can then be checked by the authority with the jurisdiction to approve its financing. Feedback loop mechanisms must be included to ensure communication of report results along the actor-chain involved in planning in order for potential improvements to be considered [21]. Approval of the mosquito control plan then would lead to a public procurement process for both PCO and QC operators, which should be performed in a timely manner with regard to the beginning of the mosquito-breeding season. This will maximize both effectiveness and efficacy of subsequent interventions, always in accordance with the public’s interests.

In Europe, depending on the country, mosquito control programs, as public health interventions, constitute a service provided by either public/NGO (not-for-profit) or private (for-profit) organizations. Transparency should be ensured throughout this process and a binding contract must be signed based on specific criteria. For example, if treatments are made by a PCO identified by a public tender, the QC activities should be well described in the contract in order to make the PCO aware of its duties and possible penalties.

The QC (i.e., the evaluation of whether the goal(s) of the program have been achieved) is likely to be the most challenging part of quality management. This is because a list of unpredicted factors, such as weather conditions, may dramatically affect mosquito population growth. In addition, internal aspects of the whole operation, such as delays in concluding procurements, shortage of funding and other issues may affect the outcome of the operation. Regardless of the difficulties, the assessment of the efficacy of the mosquito control program should be a central part of QC, should be always included, and could provide important feedback for future activities.

A variety of tools and quality indicators (see below) can be used in a thorough QC procedure to be performed by an independent/external evaluator. Intermediate short reports should be regularly submitted to funding authorities and respective administrators in order to take prompt appropriate corrective actions if needed. A final QC report should be produced at the end of the mosquito season, which should be communicated along the stakeholders/actors responsible for the identification of potential gaps and promotion of improvement actions. Intermediate short reports to be submitted to funding authorities and respective administrations are also advisable.

Enrollment of the public could be considered to enhance both transparency and provide public administrative authorities responsible for mosquito control programs with feedback on the general satisfaction levels of the residents in an objective way [22].

## 4. Tools and Recommendations for Implementing QA and QC

QA and QC processes may be implemented at different geographic scales and involve a range of actors. First, at the national level, legislation to render quality management compulsory and an integral part of the mosquito control program needs to be established or updated. At the regional or local (municipality) scale, authorities should ensure that QA and QC processes are explicitly included in procurements and activities related to mosquito control.

To promote objectivity and fair evaluation, all QA and QC processes should be conducted by an independent/external body of actor(s), who are not connected with those making decisions and/or conducting activities. This is crucial, to avoid a conflict of interest with the authorities assigning and/or carrying out control programs.

A list of tools to execute the QA is outlined in Table 3 (also see Table 1 for additional elements). The outcome of a thorough QA should be a timely report to the contracting authorities. The report should identify weak points, provide a frank evaluation of the mosquito control plan and suggest improvement measures/modifications that should be considered before the implementation. A summary of this QA may become publicly available and could be posted online on selected portals. Additional aspects, such as economic (feasibility of the program objectives, cost-effect aspects), environmental (e.g., avoid treatment of sensitive areas) and social (actions compatible with general public’s perceptions) aspects should also be considered by the funding agents separately from operational actions.

The QA report is an important document that provides the foundation for the QC. Hence, all tools and approaches required for the implementation of QC (i.e., toxicology of the products used, georeferencing of the treatments, door-to-door visits, timing, schedule and localization) should be listed in the QA report, and a clear procedure for execution should be given. In addition, the QA report should include a section that describes the procedures that should be followed if an amendment is necessary. This means that the QA report is a dynamic (“live”) document that can be updated regularly, considering and incorporating new information on mosquito activity (e.g., new IMS detected) and vector borne disease outbreaks within the area under control [23,24]. Any advance in mosquito control methods and approaches can be incorporated through this process [19,25]. Quality assurance should be concluded well before opening a procurement and well before the mosquito season begins, so that there is time to allow program adaptation according to QA results. Decision making processes (e.g., flow of information, latency of decision making, etc.) at all levels of hierarchy should be subjected to quality evaluation as well and this can become part of QA.

As stated earlier, QC encompasses two elements; first to ensure that all mosquito control activities have been properly conducted and second to evaluate whether the goals of the program objectives (i.e., nuisance reduction) have been met. Based on documents provided by the operator and the QA report, QC actors should perform unbiased monitoring, evaluating if all program activities have been conducted appropriately. Quality control monitoring methods and approaches should be adapted to the type of activities employed. For example, surveillance activities using traps can be validated by checking the presence, condition and function of traps, as well as the frequency of servicing and the quality of data generated. In control activities targeting larvae, mortality rates in treated breeding sites, tests of killing capacity of water sampled from treated ponds in controlled laboratory conditions, as well as adult emergency rates from treated and non-treated sites should be included. Quality control will greatly benefit if timely announcement of treatment planning and real time reporting of activities by service provider (e.g., PCOs) and by the QC actor are conducted.

To evaluate the outcome of the control activities, indicators regarding mosquito abundance and nuisance on humans should be employed. A meticulously designed system to monitor spatial and temporal trends of mosquito populations in treated and, if possible, non-treated control areas (otherwise data on mosquito population before and after treatment could be used) should be established. This system should allow a thorough statistical analysis of mosquito population dynamics and the quantification of possible external and stochastic events. Gathering and archiving historic data of mosquito populations in an area can provide the foundation for evaluating the efficacy of implemented actions in a contemporary and future timeframe. Likewise, a thorough analysis of nuisance reported (e.g., gathered through a mobile phone app, Table 3) provides an additional tool to evaluate whether the goals of the mosquito control program have been achieved. A detailed QC report should be available to all involved stakeholders and operators, providing a major input for future operation and QAs. A summary of the results of the QC should be made publicly available allowing future analyses/comparison of cost/effectiveness of the different control approaches.

## 5. Obstacles and Challenges of QA and QC Programs

The initial action to start an effective and sustainable mosquito control program is the establishment of a mosquito management board able to identify the most appropriate mosquito surveillance and control objectives according to the social and political context, the available human and material resources and the local entomological and epidemiological scenario. Ideally, a cost-benefit analysis on the mosquito management program should be conducted by independent experts to evaluate the convenience of implementing the program [26].

An effective engagement and collaboration between scientific, technical and political institutions at a local, regional and national level reinforces decision maker’s awareness and generates improved and sustainable cost-effective mosquito management activities, QA procedures and QC protocols. Quality control activities should have a dedicated budget and should be clearly defined with appropriate SOPs to assist the responsible authorities while assuring transparency.

To maintain effective and sustainable mosquito control projects, it is also important to identify, harmonize and regularly update mosquito surveillance and control guidelines at a national and international scale, implementing at the same time the most appropriate QM process. The possible advent of new mosquito genetic control strategies currently available for tropical mosquito species is a clear example of a future challenge in the identification of shared QM procedures to assure the achievement of appropriate entomological and epidemiological results.

The implementation of an effective QM process alone may not be able to guarantee the effectiveness of the entire mosquito control plan. However, it is expected to provide a wealth of systematically collected data that can be (a) used to improve some aspects of existing programs or (b) considered in planning future programs. The reduced availability of effective conventional vector control strategies and biocides, the increasing cases of resistance to insecticides and the necessity to reduce the impact of chemicals on the environment and non-target species, reinforce the importance of adoption of routine QC activities in all vector control programs.

The establishment of consensual thresholds for larval and/or adult population reduction under different entomological and epidemiological scenarios is quite challenging and should often be case specific. However, it is essential to develop practical methods to determine the rate of success of the mosquito control program [27]. Therefore, the QM process has to be linked to these indices, which are not easy to set and have to use QC tools capable of evaluating the achievement on these parameters.

Community participation is particularly relevant in the case of control programs targeting invasive urban *Aedes* mosquito species, such as *Ae. albopictus,* in order to prevent reduced accessibility to private properties for surveillance and control activities and an overall low participation in source reduction campaigns. The difficulties in implementing effective public engagement strategies can seriously affect the QA process of mosquito control programs especially when targeting invasive urban *Aedes* mosquito species.

QA and QC are both complex and demanding processes that might be proportionally “expensive” in control programs of medium/small size. However, since it is absolutely essential for all programs regardless of their size, an adjustment should be done for smaller surveillance and control programs that does not jeopardize their main goals.

## 6. Conclusions and Perspectives

Quality management, including quality assurance and quality control, should become an essential pillar of any mosquito control campaign regardless of whether the target of the campaign is nuisance reduction, disease prevention, outbreak control or eradication/containment of an invasion event. This is strongly supported by a clear majority of medical entomologists, public health officials and vector management professionals participating in a recent survey within the frame of the AIM-COST project. Interestingly, the specific role of QA and QC is unclear to many of these professionals and therefore requires consideration and inclusion in all legal and other related documents.

Herein, we used the term “quality management” as the overall monitoring of the control interventions. “Quality management” is divided into “quality assurance” to verify the operational approaches followed and the efficacy of the procedures, and “quality control”, which deals with the implementation of the planned actions and the validation of the outcome that is directly related with the aims of the project. In addition, QM should include processes to evaluate decision making at all levels of administration and different actors. Significant delays in control program implementation, especially for routine treatments, are frequently due to deferrals in decision making, procurements and “negotiations”, at different levels of administration. Delays in the implementation of the mosquito control programs negatively affect their efficacy, since the initial stage of mosquito population development is not managed in a timely manner.

There are generic and specific subjects that require special approaches when a QM strategy is adopted. Herein, we outline the basic procedures and elements (see Table 1), generic steps, tools and approaches (see Table 2) of QM, leaving the more detailed technical aspects to other more technical future contributions. As indicated earlier, different types of control programs (e.g., application of chemical synthetic insecticides or SIT approaches) require different QM tools and approaches.

Four elements need to be specifically stressed:

1. The need for an independent/external QM unrelated to those making decisions or implementing the actions. Often, quality checks are performed either by the funding organizations or the operators. These activities cannot replace the external evaluation of the whole program.
2. The transparency of the whole operation is assured by independent evaluation and frequent communication of the evaluation reports to the public. This facilitates a strong public support and involvement in mosquito control campaigns.
3. Ensuring an appropriate budget within each mosquito control program is dedicated to QA and QC processes is fundamental for achieving the above goals. Hence, a proportion of the budget should be allocated for this purpose—anticipated in the initial proposal—and specific procurements for independent experts should be included.
4. Implementation of the periodic and final reporting of evaluation of the efficacy of actions. It is important to identify whether the program goals have been met and eventually what corrective measures need to be taken in the next mosquito control season.

In conclusion, a QM system should not be seen only as a monitoring mechanism to identify weaknesses and define responsibilities but mainly as a dynamic tool to increase efficacy of mosquito control programs, at a cost effective, and environmentally and socially sound manner. As such, QM should become a major instrument to improve on-going and future programs.

## Figures and Tables

**Table 1:** Procedures, methods and indicators for quality assurance (QA) and quality control (QC) of mosquito surveillance and control programs in Europe.

Procedure	Methods	Quality Assurance (QA)	Quality Control (QC)
Long term surveillance	Egg monitoring (ovitraps)	Evaluate the sampling plan; estimate the trap coverage or number of “sampling units” and the trap management plan; estimate the monitoring effort and cost	Assessment of trap/sampling performance; timeliness and quality of data reporting (e.g., databases, maps)
	Larval monitoring (water netting or dipping)		
	Adult monitoring (host seeking, adult resting, adult trapping)		
Insecticide treatment	Biocides (larvicides)	Mapping of potential breeding sites; environmental and health safety; genotypic and phenotypic insecticide resistance; estimate the treatment effort and economic cost and contrast them against planning	Assessment of live larvae in breeding sites and/or adult emergence; analysis of larvae population dynamics and growth; biocides efficacy and persistence analysis
	Biocides (adulticides)		Measures of adult mortality and population density and dynamics
Source reduction (public and private areas)	Removal of mosquito breeding sites, prevention of water accumulation, avoidment of mosquito access, introduction of natural enemies (e.g., fishes, copepods)	Identification of areas with high potentially removable breeding sites (e.g., landfill, ponds); estimate the reduction effort and cost and contrast them against planning	Number and/or proportion of removed breeding sites out of initial estimates; presence of nets on barrel or rainwater reservoirs; absence of water in outdoor containers; presence of natural enemies inside ornamental or permanent ponds
	Raising of public awareness (communication campaigns)	Identification of target audience (e.g., nurseries, used tires, gardens); estimate the cost of communication campaign	Assess the success of communication campaign; questionnaire post interventions (KAP studies)
	Autocidal treatments (e.g., autodissemination)	Estimate the number of breeding sites in private areas; environmental and health safety; estimate the treatment effort and cost	Artificial breeding site sentinel; efficacy analysis and persistence; estimate the success of breeding sites reduction
Adult mass trapping	Lethal ovitraps, sticky- gravid trap, BG-trap	Estimate of trap coverage in target habitat; evaluate the trap management plan; estimate the cost-effectiveness	Number of traps deployed, and mosquito trapped; monitoring of mosquito density and dynamics
Male-releases (e.g., *Aedes albopictus*)	Sterile Insect Technique (SIT)	Regulation; target mosquito population size and dynamics; estimate the dose of sterile males and frequency of releases; coverage (area to be treated); examine the quality profile of males planned to be released; estimate the cost-effectiveness	Field induced sterility and population suppression; impact on the epidemiological risk assessment; field competitiveness indices of release males; dispersion of released males; routine quality test of released males
	*Wolbachia* infected males (Incompatible Insect Technique, IIT)		

**Table 2:** A framework for the application of QA and QC in mosquito control programs considering the operational phase, actions and procedures, and dissemination level.

Phase	Action/Procedure	Input/Justification	Quality Management	Dissemination Level
Exploration	Necessity determined	Public pressure, historic elements, estimated risk, scientific opinion	Evaluation of decision making—delays may dramatically affect the efficacy of implemented actions	Report to responsible officials. Part of the QA
	Mandate issued	National, regional or local authorities	Validity of the demand. Clearly state the goals of the project	Public report. Part of the QA
Planning	Mosquito management plan developed	Set of actions, spatial and chronological extend of the operations, environmental and social aspects of the operations, budget	QA based on experts’ opinion and analyses	QA confidential report to decision maker
	External plan evaluation developed	Experts panel, filling a quality assurance report		Public summary report of the QA
	Approval concluded and public procurement developed	Legal authority, funding provider	QA report included in the approved mosquito control plan	
Operation	Procurement advertised	Respective directorate of the authority	Based on approved plan	Public. Clear criteria
	Selection of operator concluded	Respective directorate of the authority	Competence, experience and cost-effectiveness	Public
	Program implementation started	Detailed plan of activities, including, schedule, spatial and temporal plan, periodic report on executed activities and effectiveness evaluation	QC of all performed activities. Communicating periodic reports to responsible authorities. Suggest mitigation measures	QC confidential period report to funding agency. Publicly available summary report
Evaluation	The outcome of the whole program contrasted against goals	Final report on achievements of the program’s target	Periodic report of frank evaluation of efficacy of actions implemented. Final report on whether the project goals have been met	QC confidential period report to funding agency. Publicly available summary report

**Table 3:** Tools and approaches for implementing a QM system; quality assurance (QA) and quality control (QC).

Quality Management	Tools/Approaches	Purpose
Quality Assurance (QA)	Mapping the target territory characteristics	Define social sensitive areas (e.g., schools, hospitals)
		Identify environmental sensitive areas (e.g., wetlands, Natura 2000, used-tire storage areas); identify habitats of sensitive non-target organisms
		Visualize the area of operation and analyse relevant landscape elements (e.g., breeding sites)
	Database of tools used	Registered biocides: include all available information regarding toxicological reports and side effect, indications of resistance
		Sterile mosquitoes in autocidal control programs: levels of sterility, method of inducing sterility, sex ratio of released individuals (SIT, IIT). In programs including an IIT component aspects such as sex ratio, *Wolbachia* strain etc.
	Economic analysis	Assess the cost effectiveness and economic sustainability of the program. Conduct a cost-benefit analysis.
	Questionnaire	Assess social perception of the mosquito control necessity and acceptance of the considered methodology.
	Environmental risk	Evaluation of the environmental risk, assurance of environmental compatibility.
Quality Control (QC)	Mapping (data outcome)	Real time reporting.
		Documentation of executed actions.
		Evaluation of side effect on sensitive areas.
	Web-based platform	Real time reporting to involved parties e.g., GPS monitoring of operational units (restricted area).
		Communicating outcome of QC to public (open area).
	Mosquito sampling using adult trapping, ovitraps, breeding sites larvae/pupae density	Estimate mosquito population density before and after treatments and seasonally.
	Mobile application to report nuisance perception	Estimate social response.
	Thorough analysis of spatial and temporal trends of mosquito population densities.	Determine the result of interventions and identify possible stochastic effects.
	Inclusion of control areas (untreated areas)	Contrast population dynamics with treated areas (if possible, included in QC, after approval from the respective ethics committee).
	Assessment of target mosquitoes’ sensitivity to insecticides	Prevent control failures, develop and adopt resistance management plan.
	Estimation of environmental quality indexes	Assessment of possible side effects of mosquito control interventions on non-target organisms and the environment at a whole.

## Data Availability

Not applicable.
